# Polyphenolic natural products as photosensitizers for antimicrobial photodynamic therapy: recent advances and future prospects

**DOI:** 10.3389/fimmu.2023.1275859

**Published:** 2023-10-31

**Authors:** Xiaoyun Wang, Lian Wang, Reza Fekrazad, Lu Zhang, Xian Jiang, Gu He, Xiang Wen

**Affiliations:** ^1^ Department of Dermatology, West China Hospital, Sichuan University, Chengdu, China; ^2^ Laboratory of Dermatology, Clinical Institute of Inflammation and Immunology, Frontiers Science Center for Disease-Related Molecular Network and State Key Laboratory of Biotherapy, West China Hospital, Sichuan University, Chengdu, China; ^3^ Radiation Sciences Research Center, Laser Research Center in Medical Sciences, AJA University of Medical Sciences, Tehran, Iran; ^4^ International Network for Photo Medicine and Photo Dynamic Therapy (INPMPDT), Universal Scientific Education and Research Network (USERN), Tehran, Iran

**Keywords:** polyphenols, natural products, photodynamic therapy, photosensitizers, antibacterial

## Abstract

Antimicrobial photodynamic therapy (aPDT) has become a potent contender in the fight against microbial infections, especially in the context of the rising antibiotic resistance crisis. Recently, there has been significant interest in polyphenolic natural products as potential photosensitizers (PSs) in aPDT, given their unique chemical structures and inherent antimicrobial properties. Polyphenolic natural products, abundant and readily obtainable from natural sources, are generally regarded as safe and highly compatible with the human body. This comprehensive review focuses on the latest developments and future implications of using natural polyphenols as PSs in aPDT. Paramount polyphenolic compounds, including curcumin, hypericin, quercetin, hypocrellin, celastrol, riboflavin, resveratrol, gallic acid, and aloe emodin, are elaborated upon with respect to their structural characteristics, absorption properties, and antimicrobial effects. Furthermore, the aPDT mechanism, specifically its targeted action on microbial cells and biofilms, is also discussed. Polyphenolic natural products demonstrate immense potential as PSs in aPDT, representing a promising alternate approach to counteract antibiotic-resistant bacteria and biofilm-related infections.

## 1 Introduction

**Figure 1:**
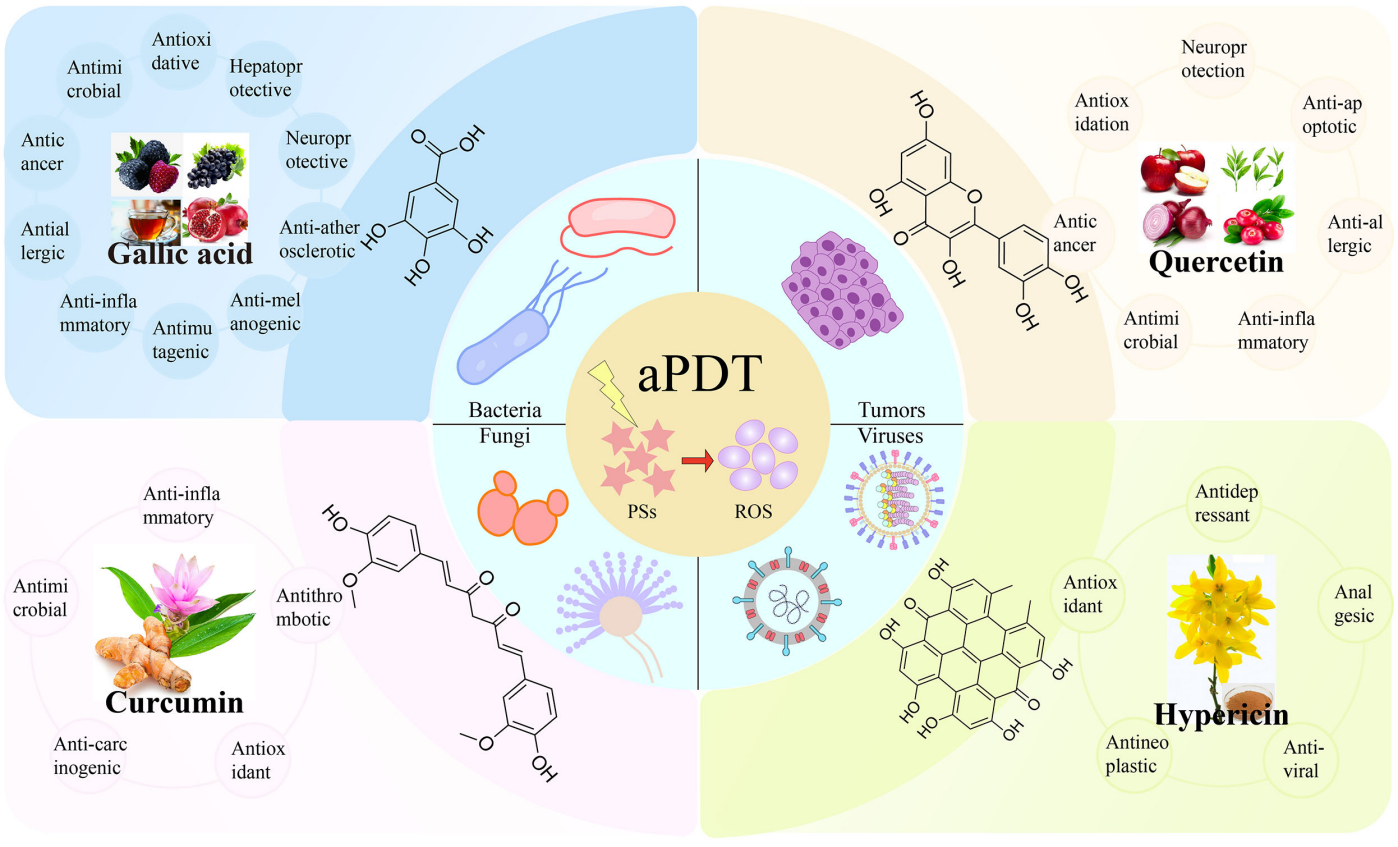
The representative polyphenolic natural products as photosensitizers for antimicrobial photodynamic therapy.

The pervasive phenomenon of antimicrobial resistance (AMR) in a broad array of pathogenic microorganisms presents a grave and pressing concern for global health and developmental progress (1). Multidrug resistance (MDR) in bacteria culminates in hundreds of thousands of deaths annually (2), underscoring AMR’s role as a profound international health concern (3). Concurrently, the limited availability of effective drugs for fungal infections and the rising resistance to these drugs have led to a distressingly high mortality rate (4–6). Additionally, the unprecedented emergence of SARS-CoV-2 has introduced a global threat to human life and health. The severe implications of antimicrobial resistance on human health and economic systems necessitate the accelerated development of innovative strategies to counteract this formidable issue effectively (7, 8). As shown in **Figure 1**, in response to this, there has been a surge of research interest dedicated to developing alternative solutions to combat antimicrobial resistance, such as cationic polymers, peptidoglycans, metal nanoparticles, nanocarriers, photodynamic therapy (PDT), and photothermal therapy (PTT) (9, 10).

PDT is a therapeutic modality that employs low-energy light to activate photosensitizers (PSs) for both diagnostic and therapeutic purposes. Antimicrobial photodynamic therapy (aPDT), a specific application of PDT, serves as a chemical treatment method to control infections caused by bacteria, fungi, and viruses. As a potent and promising alternative, aPDT strives to mitigate the proliferation of pathogenic microorganisms, encompassing both gram-positive and gram-negative bacteria, fungi, viruses, and parasites. This is achieved by curtailing microbial growth, preventing biofilm formation, and potentially resolving antibiotic resistance issues (11, 12). One notable advantage of aPDT is its noninvasive or minimally invasive nature, which enables a targeted approach primarily against the microorganisms, sparing animal tissue cells from unnecessary damage. This relatively simple and selective approach ensures effective pathogen elimination while minimizing harm to the host (13). The fundamental components of aPDT include the light, PSs, and ambient oxygen. Independently, these elements are benign, but their amalgamation can render a potent antimicrobial effect. This process entails the use of a PS, which, when activated by a particular wavelength of light in the presence of oxygen, generates a copious amount of reactive oxygen species (ROS). These ROS, in turn, interact with multiple targets within microbial cells, inducing the oxidation of biomolecules and ultimately causing cell death.

PSs play an instrumental role in aPDT because they are responsible for absorbing light energy. Various synthetic compounds such as tetrapyrrole macrocycles (porphyrins, phthalocyanines), heterocyclic compounds (methylene blue, toluidine blue O), indocyanine green, and psoralens have been extensively studied for their antibacterial potency in aPDT (14–16). In contrast to synthetic compounds, natural products are generally imbued with more complex chemical structures, granting them unique capabilities in moderating physiological processes and contending with external threats. Derived from natural sources such as plants, animals, and microorganisms, these products acquire unique chemical structures through prolonged evolutionary processes. These structures can engage with molecular entities within organisms, thus intervening in and regulating numerous physiological processes. Among these natural products, polyphenols represent a noteworthy class of compounds found abundantly in various plant-based products, such as vegetables, fruits, seeds, and legumes. Characterized by a series of molecules bearing one or more phenolic rings (17, 18), polyphenols frequently exhibit a diverse array of biological activities, including antioxidant, anticancer, antibacterial, antiviral, and anti-inflammatory properties, which render them potent candidates for the treatment of infections and other diseases (19–21). The significance of polyphenolic natural products in aPDT is underscored by their traditional role as a source for modern drug discovery, offering potential drug leads due to their unique structures, diverse chemical and biological properties, and antimicrobial and anti-inflammatory characteristics (22, 23). Consequently, polyphenolic natural products as PSs have gained considerable attention in the field.

**Scheme 1:**
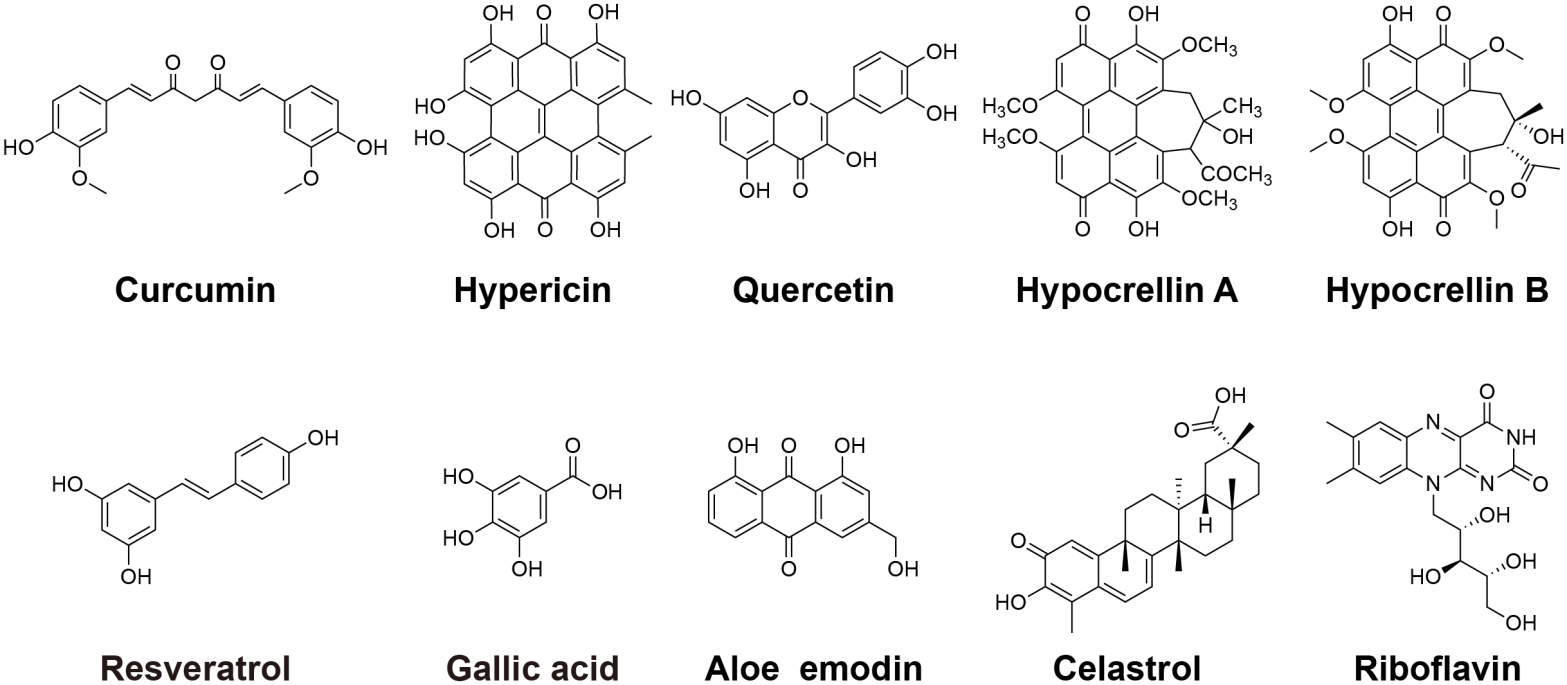
The chemical structures of polyphenols.

This review focuses on recent advances and future prospects of aPDT for treating microbial infections, with a specific emphasis on the application of polyphenolic natural product PSs (**Scheme 1**). The unique properties and promising potential of these compounds in combating infections warrant further exploration and development to identify effective therapeutic interventions.

## 2 Polyphenolic natural PSs

### 2.1 Curcumin

Curcumin (CUR), a natural polyphenol extracted from the dried rhizomes of the ginger plant turmeric (*Curcuma longa L.*), has a long history of culinary, traditional medicinal, cosmetic, and herbal supplement use (24). Chemically, curcumin is a diarylheptanoid, a polyphenol, with beta-diketone and enone functionalities, and its structure is related to a dimer of ferulic acid (25). Natural curcumin consists of three distinct curcuminoids: curcumin, demethoxycurcumin (DMC), and bisdemethoxycurcumin (BDMC) (21). The compound demonstrates a broad spectrum of pharmacological effects, including anti-inflammatory, antimicrobial, anticarcinogenic, antioxidant, and antithrombotic activities (26, 27). Curcumin is known for its safety, efficacy, and environmentally friendly characteristics (28). It has also been extensively investigated as a highly effective PS in the field of photodynamic therapy due to its broad absorption range between 300 and 500 nm and its nontoxicity in cell culture models and animal studies (29). Due to its favorable properties, curcumin has been extensively researched for its therapeutic potential and supportive care in clinical conditions such as breast cancer, multiple myeloma, non-small cell lung cancer, and depression (30–33).

As a natural compound, curcumin has been widely investigated as a PS in aPDT. For instance, Li et al. demonstrated the effective eradication of *Bacillus subtilis* (*B. subtilis*) through curcumin-mediated PDT by inducing an imbalance in the cellular redox state, causing DNA damage and disrupting membrane structures (13). Wang et al. demonstrated that curcumin (25 μM)-mediated aPDT could inhibit 5 log CFU/ml of *Staphylococcus saprophyticus* (*S. saprophyticus*) with the irradiation parameters (430-470 nm, 4.32 J/cm^2^ 10 min) in food production (34). Abdulrahman et al. concluded that curcumin-mediated aPDT inhibits the biofilm formation by 70% of *Pseudomonas aeruginosa* (*P. aeruginosa*) with 10 J/cm^2^ laser light and 6.75 mM of curcumin (35). However, the use of curcumin in aPDT is currently limited to local applications on superficial wounds, such as the skin and oral cavity, primarily due to its absorption of blue light within the light spectrum (300-500 nm), which has restricted tissue penetration capabilities. Muniz et al. demonstrated that curcumin (100 µg), as a PS being activated *ex vivo* by LED (450 nm, 13.5 J/cm^2^), effectively controlled *Staphylococcus aureus* (*S. aureus*) infections in mice with type 1 diabetes mellitus (36). Méndez et al. found that curcumin-mediated aPDT effectively reduced the viability of microbial cells and compromised the vitality of intact biofilms of infected dentin caries microcosms (37). Moreover, curcumin-mediated aPDT has shown efficacy against various pathogens, including *Escherichia coli* (*E. coli*, inactivated up to 3 log CFU/mL), *Listeria innocua* (*L. innocua*, inactivated more than 5 log CFU/mL) in food systems (38), *Propionibacterium acnes* (*P. acnes*, inhibition ratio was 100%) associated with acne vulgaris (39), significantly decreased planktonic *Streptococcus mutans* (*S. mutans*) and *S. mutans* biofilm (2 log10 CFU/mL reductions) in dental caries (40, 41), complete kill of *Aggregatibacter actinomycetemcomitans* (*A. actinomycetemcomitans*) (42), methicillin-resistant *S. aureus* biofilm (2.03 log10 CFU/mL reductions) (24), and fungi such as *Candida albicans* (*C. albicans*, 1 log reductions) and other stains of the *Candida* spp. (43) **Table 1** for a detailed description of the application of polyphenols as PSs in aPDT.

**Table 1:** Polyphenols as PSs for aPDT.

Polyphenols	The absorption range/peak	Light type and parameters (wavelength, power/power density, irradiation time) ^*^	Microorganisms	Concentration and incubation time of PSs	Efficacy	Reference
Curcumin	420-470 nm	Blue LED, 470 nm, 120 W, 6 min	*B. subtilis*	50 µM, 15 min	Effectively kill	(13)
		Blue LED, 430-470 nm, 4.32 J/cm^2^ 10 min	*S. saprophyticus*	25 µM, 15 min	5 log CFU/ml reductions	(34)
		405 nm light, 10 J/cm^2^ 26 s	*P. aeruginosa*	6.75 mM, 10 min,	4.62 log_10_ planktonic cell reductions	(35)
		Blue LED, 450 nm, 13.5 J/cm^3^ 180 s	*S. aureus* (MRSA)	100 µg/mice, NR	Effectively control the burden of MRSA in type 1 diabetes mellitus mice	(36)
		Blue LED, 455 ± 30 nm, 75 J/cm^3^ 1870 s	Intact microcosm biofilms of dentin caries	NR	Reduced substantially the vitality of intact microcosm biofilms	(37)
		UVA, 320-400 nm, 32 W/m^2^ 5 min	*E. coli O157:H7*	1~10 mg/L, 5 min,	Inactivate up to 3 log CFU/mL	(38)
		UVA, 320-400 nm, 32 W/m^2^ 5 min	*L. innocua*	1~10 mg/L, 5 min,	Inactivate more than 5 log CFU/mL	(38)
		LED, 410-510 nm, 0.09 (0.18) J/cm^2^, 0.5 (1) min	*P. acnes*	1.56~100 µM, NR,	Inhibition ratio was 100%	(39)
		LED, 405 nm, 25.3 J/cm^2^, 300 s	*S. mutans*	10^4^ ng/mL, NR,	Significantly decreased	(40)
		Blue Light, 385-515 nm, 14.6 J/cm^2^, 60 s	*S. mutans* biofilm	0.10wt% CUR loading on resin physicochemical, 6 h or 24 h	2 log_10_ CFU/mL reductions	(41)
		LED, 420-480 nm, 16.8 J/cm^2^, 1 min	*A. actinomycetemcomitans*	0.78 μg/mL Curcuma longa extract, 48 h	Complete kill	(42)
		LED, 450 nm, 50 J/cm^2^, 455 s	*MRSA* biofilm	80 μg/mL, 20 min	2.03 log_10_ CFU/mL reductions	(24)
		Blue LED, 450 ± 5 nm, first:10 J/cm^2^, 91 s; second: 25 J/cm^2^, 228 s	*C. albicans*	200 µg/mL, 20 min	1 log reductions	(43)
		Blue LED, 450 ± 5 nm, first:10 J/cm^2^, 91 s; second: 25 J/cm^2^, 228 s	*C. tropicalis*	200 µg/mL, 20 min	5 log reductions	(43)
Hypericin	590-595 nm	LED, 660 nm, 100 J/cm^2^, 30 s	*P. acnes* biofilms	15 µg/mL, 3 min	14.1% reductions	(44)
		BL-300 LED, 585 nm, 9.2 J/cm^2^, 40 min	*B. cereus*	10^-7^ M, 60 min	4.4 log CFU/mL reductions	(45)
		LED, 590 nm, 48 J/cm^2^, 10 min	*S. aureus*	1 µg/mL, 5 min	6.3 log killing	(46)
		LED, 590 nm, 48 J/cm^2^, 10 min	*E. faecalis*	1 µg/mL, 5 min	6.5 log killing	(46)
		LED, 590 nm, 48 J/cm^2^, 10 min	*E. coli*	1 µg/mL, 5 min	6.2 log killing	(46)
		LED, 590 nm, 48 J/cm^2^, 10 min	*P. aeruginosa*	1 µg/mL, 5 min	0.7 log killing	(46)
		LED, 602 ± 10 nm, 18 or 37 J/cm^2^, 10 min	Azole-resistant and sensitive *C. albicans*	5 or 10 µM, 5 h	5 log_10_ CFU/mL reductions	(47)
		LED, 590 nm, 150 ± 20 W/m^2^,3 h	Ampicillin-resistant *P. aeruginosa*	10 μM + ampicillin (100μg/mL), 30 min	3.4 log reductions	(48)
		LED, 590 nm, 150 ± 20 W/m^2^,1 h	*C. albicans*	10 μM, 30 min	4.8 log reductions	(48)
		LED, 590 nm, 16 J/cm^2^, 10 min	*S. aureus* biofilms	0.5 µg/mL + 10 mg/mL acetylcysteine, 5 min,	5.7 log killing	(49)
Quercetin	405nm	Xenon lamp, 365 nm, 70 mW/cm^2^, 240 s	*E. coli*	500 mM, 2 h or 6 h,	No effect	(50)
		Xenon lamp, 365 nm, 70 mW/cm^2^, 240 s	*S. aureus*	500 mM, 2 h or 6 h	Total death	(50)
		Blue laser, 405 ± 10 nm, 150 mW/cm^2^, 60 s	*S. mutans* biofilms	64 µg/mL, 5 min,	4 log_10_ CFU/mL reductions	(51)
		LED, 405 nm, 80 J/cm^2^, 68 min 21 s	*E. coli* O157:H7	75 µM, 68 min 21 s	6.20 log reductions	(52)
		LED, 405 nm, 80 J/cm^2^, 68 min 21 s	*L. monocytogenes*	75 µM, 68 min 21 s	>7.55 log reductions	(52)
		LED, 435 ± 10 nm, 300-420 J/cm^2^, 5 min	*A. baumannii* biofilms	500 µg/mL, 2 h	40.8% reductions	(53)
Hypocrellin A	400-700nm	Incandescent lamp,400-780 nm, 1128 lux, 30 min	*C. albicans*	1.0 μg/mL, 30 min	Approximately 50% reductions	(54, 55)
		Laser, 470 nm, 100 mW/cm^2^, 30 min	*C auris*	With polylactic acid, 30 min	>99.9% mortality	(56)
		Laser, 470 nm, 100 mW/cm^2^, 30 min	Multidrug-resistant *Candida* spp.	12.5 μg/mL with polyethylene glycol, 30 min	Completely kill	(57)
		NR, 470 nm, 90 mW/cm^2^, 60 min	Methicillin-resistant *S. aureus*	1.38 mg/L with mPEG-PCL, 24 h	Minimum bactericidal concentration	(58)
Hypocrellin B	450-550nm	Xenon lamp, 400-780 nm, 72 J/cm^2^, 15 min	*C. albicans*	100 µM, 30 min	No viable cells	(59)
		Xenon lamp, 400-780 nm, 72 J/cm^2^, 15 min	Azole-sensitive clinical isolate of *C. albicans*	100 µM, 30 min	6.01 log_10_ reductions	(59)
		Xenon lamp, 400-780 nm, 72 J/cm^2^, 15 min	Azole-resistant clinical isolate of *C. albicans*	100 µM, 30 min	7 log_10_ reductions	(59)
		LED, 460 ± 20 nm/645 ± 20 nm, 24 J/cm^2^, 3 min	Drug-resistant *P. aeruginosa*	10 µM (HB: La^+3^)^&^, 5 min	5 log reductions	(60)
Resveratrol	200-330nm	Blue LED,450 ± 20 nm/, 54 J/cm^2^, 5 min	*S. aureus*	2 mg/mL, 5 min,	Approximately 75% reductions	(61)
Gallic acid	273nm	UVA-light, 2646 ± 212 μW/cm^2^, 15 min	*E. coli O157:H7*	10 mM, 15 min	4.95 ± 0.19 log CFU/mL reductions	(62)
		UVA-light, 3.2 ± 0.2 mW/cm^2^, 30 min	*E. coli O157:H7*	1 mM with 5 mM lactic acid, 30 min	4.7 ± 0.5 log CFU/ml reductions	(63)
		LED, 400 nm, 80 mW/cm^2^, 15 min	*S. aureus*	4 mmol/L, 15 min	>5 log reductions	(64)
Aloe emodin	250nm, 284nm, 430nm	Xenon lamp, 435 ± 10nm, 96 J/cm^2^, 20 min	Multidrug-resistant *A. baumannii*	100 µM, 20 min	4.50~6.89 log_10_ reductions	(65)
		Xenon lamp, 400-780 nm, 24 J/cm^2^, 5 min	*C. albicans* (a standard strain)	5 µM, 30 min	5.84 log_10_ reductions	(66)
		Xenon lamp, 400-780 nm, 24 J/cm^2^, 5 min	Azole-sensitive *C. albicans*	5 µM, 30 min	5.56 log_10_ reductions	(66)
		Xenon lamp, 400-780 nm, 24 J/cm^2^, 5 min	Azole-resistant *C. albicans*	5 µM, 30 min	4.69 log_10_ reductions	(66)
		Xenon lamp, 435 ± 10nm, 72 J/cm^2^, 30 min	*T. rubrum* (control strain)	1 μM, 2 h	Decreased survival rate to 17.10%	(67)
		Xenon lamp, 435 ± 10 nm, 72 J/cm^2^, 30 min	*T. rubrum* (clinical strain)	1 μM, 2 h	Decreased survival rate to 18.63%	(67)
		Xenon lamp, 400-780 nm, 96 J/cm^2^, 20 min	*Malassezia furfur*	10 μM, 30 min	No viable cells	(68)
Celastrol and *T. wilfordii extract*	425nm	LED, 660 nm, 120 ± 20 W/m^2^, 15 min	*S. aureus*	20 µg/mL (TWE), 30 min	3.3 log reductions	(69)
		LED, 660 nm, 120 ± 20 W/m^2^, 10 min	MRSA	20 µg/mL (TWE), 30 min	3.4 log reductions	(69)
		LED, 660 nm, 120 ± 20 W/m^2^, 30 min	*C. albicans*	20 µg/mL (TWE), 30 or 60 min	2.0 log reductions	(69)
Riboflavin	270 nm, 366 nm, and 445 nm	LED, 365 nm, 30 J/cm^2^, 1 h	*S. aureus*, *P. aeruginosa* *E. coli*	0.1 mg/mL with PEG, 15 min	Approximately 4 log reductions	(70)
		LED, 365 nm, 30 J/cm^2^, 1 h	*S. typhimurium*, Coliphage	0.1 mg/mL with PEG, 15 min	Approximately 3 log reductions	(70)
		Blue light, 460 nm, 80 mW/cm^2^, 10 min	*S. aureus, E. coli*, MRSA	100 µL (Riboflavin-loaded supramolecular hydrogels), NR,	Inhibition ratio over 99.999%	(71)

NR, not reported; *The irradiation frequency is 1 without special explanation; ^&^Hypocrellin B with lanthanide ions; MRSA,: methicillin-resistant *Staphylococcus aureus*; LED, light-emitting diode; UVA, ultraviolet A; CUR, curcumin; mPEG-PCL, methoxy poly (ethylene glycol)-block-poly(ϵ-capro-lactone); TWE, ethanolic extract of *T. wilfordii*; PEG, polyethylene glycol; CFU, colony forming units.

However, the excellent biological and pharmacological activities of curcumin are hindered by its inherent physicochemical properties, including low solubility, rapid metabolization, instability, and the presence of a negative charge state, which hampers effective contact and adhesion to the surfaces of bacteria with negative charge (72). Extensive research has been conducted to address these challenges, particularly through the exploration of an ideal nanocarrier for curcumin (73–75). Additionally, optimizing the formulation and delivery methods of curcumin-based aPDT is crucial to overcome limitations related to tissue penetration. Further research is necessary to improve the bioavailability and absorption of curcumin, maximizing its efficacy in medical and health applications.

### 2.2 Hypericin

Hypericin (HYP), a naturally occurring pigment isolated from hypericum plants of the genus *Hypericum perforatum* (commonly referred to as Saint John’s Wort), is well-known for its antidepressant, antioxidant, antineoplastic, potential antiviral and analgesic activities. It has recently been recognized as an effective and promising PS agent found in nature (44, 45). HYP, an anthraquinone derivative exhibits a high quantum yield for the generation of ROS and a slow rate of photobleaching (49, 76). It can also be synthesized from emodin, another anthraquinone derivative (77). The optical properties of HYP enable its absorbance of electromagnetic radiation within the visible spectrum range of 500-620 nm, with a peak absorbance at 595 nm. Upon light exposure, it displays strong red fluorescence, typically emitted at approximately 603 nm, contributing to its intense red fluorescence characteristics (78). HYP exhibits high lipophilicity and poor water solubility, displaying multiple absorption peaks in organic solvents within its visible spectrum, notably at 550 nm and 588 nm in ethanol. Additionally, emodin in ethanol exhibits fluorescence emission at approximately 600 nm. However, when dissolved in aqueous solutions, HYP tends to form nonfluorescent high-molecular-weight aggregates (79, 80).

Recently, there has been increasing interest in investigating the pharmaceutical potential of HYP as a PS in aPDT. Barroso et al. demonstrated effective antimicrobial activity of aPDT using HYP as a PS against *P. acnes* biofilms and highlighted its potential for clinical treatment of acne vulgaris (44). Kashef et al. investigated the high phototoxicity of HYP against *S. aureus*, *Enterococcus faecalis* (*E. faecalis*), and *E. coli* at extremely low drug concentrations. While observing minimal cytotoxic effects on cultured human fibroblast cells (46). Aponiene et al. showed efficient elimination of food-borne pathogen *Bacillus cereus* (*B. cereus*) through hypericin-based photosensitization in both *in vitro* experiments and on the surfaces of fruits and vegetables (45). Paz-Cristobal et al. confirmed the greater efficacy of HYP at lower concentrations against azole-resistant *C. albicans* (47). In a study by Alam et al., the effectiveness of PDT against *P. aeruginosa*, a gram-negative bacterium with limited PS penetration, was enhanced by combining HYP with ampicillin. This combination acted as a permeabilizing agent, disrupting the bacterial cell wall and increasing cell permeability, thereby maximizing the efficacy of PDT (48). Additionally, Kashef et al. demonstrated the efficacy of combining HYP with acetylcysteine in reducing biofilm formation and disrupting mature biofilms across various bacterial strains, notably, against *S. aureus*, a prominent pathogen (49).

Despite its desirable properties such as a high quantum yield of singlet oxygen generation, low dark toxicity, a high extinction coefficient near 600 nm, and significant inhibition of gram-positive bacterial growth, the utilization of HYP in biological applications is limited by its high lipophilicity and water insolubility in its natural form. Consequently, its potential in biopharmaceuticals is constrained, and its clinical implementation faces substantial hurdles. Therefore, the development of a delivery system is crucial to overcome these limitations. Various delivery systems, including polymeric nanoparticles and liposomes, have been extensively explored for HYP, showing promising results (76, 81–83).

### 2.3 Quercetin

Quercetin (QCT), a natural polyphenol, belongs to the subclass of flavonols, one of the six subclasses of flavonoid compounds (84). It is abundantly found in various fruits and vegetables such as apples, grapes, onions, and tomatoes, as well as beverages such as tea and red wine, nuts and honey, from different plant sources (50, 84). As a secondary metabolite, QCT exhibits a diverse array of pharmacological activities, including neuroprotection, antioxidation, antimicrobial, anticancer, anti-inflammatory, and anti-allergic and anti-apoptotic effects (50, 51). QCT demonstrates distinct absorption peaks at 380 and 258 nm (85), and its biological efficacy is significantly enhanced at micromolar concentrations when activated by light within the range of 405 ± 10 nm (51).

Despite limited research on the application of QCT as a PS in aPDT, some studies have explored its correlation and potential. One study demonstrated that QCT-mediated aPDT significantly reduced the growth of *E. coli* and *Listeria monocytogenes* (*L. monocytogenes*) in a buffer solution, indicating its potential as an antimicrobial agent against these bacteria (52). Pourhajibagher et al. utilized QCT with a light-emitting diode to effectively reduce the growth of *A. baumannii* biofilms and downregulate genes involved in the biofilm formation (53). Condat et al. developed synthetic photoactivable glycerol-based coatings incorporating QCT, which demonstrated a remarkable 99% inhibition of *S. aureus* proliferation after 2 and 6 hours of incubation under light activation (50). Another study conducted by Pourhajibagher et al. demonstrated that the synergistic combination of blue laser and low-concentration nanoquercetin can disrupt the microbial biofilm of *S. mutans* and reduce its metabolic activity (51). However, further research is necessary to evaluate the antibacterial pharmacological activity of QCT and determine its potential value in clinical applications.

### 2.4 Hypocrellins

Hypocrellins, primarily composed of hypocrellin A and B, which are perylenoquinone derivatives, are obtained from the fruiting bodies of the traditional Chinese medicine fungi *Hypocrella bambusae* and *Shiraia bambusae* (86, 87). Hypocrellins, structurally related to HYP, are predominantly lipophilic, although a few hydrophobic hypocrellin derivatives have been synthesized, with limited studies on their properties (86, 88). Structurally, hypocrellin A (HA) and hypocrellin B (HB) exhibit a high degree of similarity, differing only by the presence of a single hydroxyl group (59, 89). Hypocrellins exhibit several advantageous characteristics, including a notable quantum yield for singlet oxygen (^1^O_2_) generation, strong generation of anionic free radicals in deoxygenated environments, rapid clearance from normal tissues, minimal dark toxicity, and existence in a pure monomeric form. These exceptional attributes have led to the extensive utilization of hypocrellin as a PS in photodynamic therapy (89). In ethanol, HA exhibits three distinct absorption peaks at 581 nm, 542 nm, and 463 nm, within the visible light spectrum range of 400 - 700 nm (54). The absorption wavelength of HB ranges from 450 nm to 550 nm (90).

Hypocrellins have been extensively studied for their potential applications in treating various dermatological conditions, and viral infections, including human immunodeficiency virus (HIV), and even cancer (91). Due to their unique characteristics, such as ease of preparation and purification, high photoreactivity with low dark toxicity (92), and rapid tissue clearance, hypocrellins have garnered significant attention as novel therapeutic agents and/or diagnostic tools (87, 91). In PDT, HA plays a crucial role in anticancer treatment (93). However, research on the antimicrobial photodynamic activity of HA is limited and primarily focused on *C. albicans* (55), *Candida auris* (*C. auris*) (56, 57), and methicillin-resistant *S. aureus* (58). Nonetheless, the efficacy of HA is limited by certain characteristics, including poor water solubility, tendency to aggregate under physiological conditions, and limited absorption within the phototherapeutic window, which restricts its clinical application in PDT. To overcome these limitations, Guo and colleagues developed a self-assembled amphiphilic micelle that is sensitive to lipase, enabling efficient delivery of HA. The micelles composed of mPEG-PCL/HA demonstrated promising antimicrobial activity against methicillin-resistant *Staphylococcus aureus* (MRSA) (58). In another study, Liu et al. prepared a recyclable and light-triggered nanofibrous membrane of polylactic acid conjugated with HA and modified porous organic cages with HA for targeting *C. auris* and multidrug-resistant *Candida* species, respectively (56, 57). Similarly, research on HB primarily revolves around its antitumor and antiviral properties. Studies have revealed that HB demonstrates potent photodynamic effects against malignant tumors, human immunodeficiency virus type I (HIV-I), and herpetic stomatitis (90). In their *in vitro* experiments, Hu et al. demonstrated that HB-LED PDT triggers apoptosis in human keloid fibroblasts through the mitochondrial apoptotic pathway (89). Moreover, Hashimoto et al. found that HB-mediated aPDT exhibits promise as a viable alternative treatment for *P. aeruginosa*-infected burns, as it effectively reduces *P. aeruginosa* at the infection site, delays bacteremia, maintains lower bacterial levels in the bloodstream compared to untreated groups, and significantly increases the lifespan of mice (60). The Jan group investigated the photodynamic inactivation effects of HB on both azole-sensitive and azole-resistant strains of *C. albicans in vitro*. HB exhibited negligible dark toxicity and efficiently deactivated *C. albicans* cells in a light-dose and PS concentration-dependent manner (59). Recently, Law et al. proposed HB as a potential PS for PDT in the treatment of SARS-CoV-2 (94). These innovative approaches hold great potential for enhancing therapeutic outcomes in the treatment of microbial infections.

### 2.5 Resveratrol

Resveratrol, also known as trans-3,4,5-trihydroxystilbene, is a naturally derived polyphenolic compound and phytoalexin. It is synthesized in response to various stressors, including plant damage or microbial infections caused by bacteria or fungi (95). Resveratrol is commonly found in a variety of dietary substances, such as grapes, berries (cranberries), red wine, nuts (peanuts) and other foods (96–98). Chemically, it belongs to the stilbene family and acts as a fundamental precursor for the synthesis of other stilbenes, such as piceatannol and pterostilbene (specifically trans-3,5-dimethoxy-4’-hydroxystilbene) (96). Resveratrol presents a diverse array of biological activities, encompassing antimicrobial, antiviral, antioxidant, anti-aging, anti-inflammatory, and anticancer properties. Moreover, it has been recognized for its cardioprotective and neuroprotective attributes (99). These notable biological functions can be attributed to its unique molecular structure, which enables effective interactions with various biomolecules. Resveratrol displays a wide absorption spectrum ranging from 290 nm to 360 nm, with a peak wavelength observed at approximately 320 nm (100).

The antimicrobial activity of resveratrol has been studied extensively. Klančnik et al. reported a minimum inhibitory concentration (MIC) of 0.313 mg/ml for resveratrol against *Campylobacter jejuni* (101). In contrast, Duracka et al. found no significant bactericidal activity of resveratrol against *Enterococcus faecalis* in rabbit ejaculates (102). Li et al. discovered that resveratrol, at a concentration of 800 µg/mL, significantly inhibits the growth of *S. mutans* (96). Furthermore, Kugaji et al. demonstrated remarkable antibacterial and anti-biofilm activity of resveratrol against *Porphyromonas gingivalis* (*P. gingivalis*), a bacterium associated with gum disease (99). Dos Santos et al. were the first to establish a connection between aPDT and resveratrol, highlighting its effective inhibition of *S. aureus* when used as a PS (61). Resveratrol as a natural polyphenol compound, exhibits therapeutic potential. However, it is pertinent to acknowledge that the stability of the resveratrol can be influenced by factors such as UV radiation, pH, and temperature (103).

### 2.6 Gallic acid

Gallic acid (GA) (3,4,5-trihydroxybenzoic acid), a natural polyphenolic compound, is abundant in various plants, including trees, herbs, fruits, and nuts, as well as processed beverages such as red wine and green tea (104). Recognized for its inherent and potent biological activities, GA exhibits a diverse range of effects, encompassing antioxidative, antimicrobial, antiallergic, anticancer, anti-inflammatory, antimutagenic, anti-melanogenic, anti-atherosclerotic, neuroprotective, and hepatoprotective properties (105, 106). Its versatile applications span multiple fields, such as medicine, chemical research, pharmaceuticals, cosmetics, and the food industry (107). The polyphenolic functional groups present in GA contribute to its remarkable ability to scavenge oxygen-derived free radicals (108). Moreover, GA is commonly employed as a standard compound for quantifying phenol content using the Folin-Ciocalteu method (109). Derived from protocatechuic acid, GA serves as an intermediate in the secondary metabolism of plants (108). Structurally, GA is a phenolic acid consisting of benzene ring with a carboxyl group and three hydroxyl groups attached to it. Its formation can be obtained through the acid hydrolysis of hydrolysable tannins (110). It has the capability to absorb ultraviolet (UV) irradiation and light in the visible spectrum (111).

GA has demonstrated remarkable inhibitory effects on the motility, adhesion, and biofilm formation of *S. aureus*, *S. pyogenes*, *P. aeruginosa*, and *L. monocytogenes* (112–114). In an insightful study by Cossu et al., GA treatment combined with UV-A irradiation significantly inactivated metabolically active *E. coli* O157:H7 (62). Furthermore, De Oliveira et al. demonstrated that the synergistic combination of GA with lactic acid (LA) and UV-A was specifically effective against *E. coli* O157:H7 (63). A study conducted by Nakamura et al. investigated the antibacterial effect of GA (4 mmol/L) on *S. aureus* under LED light irradiation, resulting in a 99.9% reduction in bacteria. Notably, the authors suggest that the antibacterial action is induced by photooxidation and automatic oxidation of GA, as its individual bactericidal effect is less pronounced (64).

### 2.7 Aloe emodin

Aloe emodin (AE) is a naturally occurring anthraquinone derivative with structural similarity to HYP. It is extracted from traditional Chinese medicine (TCM) plants such as *Aloe vera*, *Rheum officinale Baill.*, *Rumex patientia Linn.*, *Cassia mimosoides L.* and *Polygonum multiflorum Thunb* (115, 116). AE shares a remarkable chemical structure resemblance to HYP, an extensively studied classical PS, and exhibits light absorption capability in the ultraviolet-visible regions. AE displays three primary absorption bands centered at 250 nm, 284 nm and 430 nm. Light sources within the blue region, including lasers emitting wavelengths of 405 nm, 430 nm, and 473 nm, as well as broadband light using suitable filters, effectively activate AE (65). The maximum absorption band of AE in the blue region makes AE-mediated PDT particularly advantageous for the treating of superficial diseases, including skin cancer, oral disorders, and ocular conditions. The singlet oxygen quantum yield (^1^O_2_) of AE was determined to be 0.57 (2) in methanol, which is marginally higher than that of methylene blue (117).

Recently, AE has gained increasing attention due to its potential applications in the treatment of various diseases. Several studies have indicated that aloin, a compound found in aloe vera, possesses various biological properties, including antiviral, antibacterial, anti-inflammatory, and hepatoprotective activities (118–120). Moreover, AE has demonstrated anticancer activity against lung squamous cell carcinoma, neuroectodermal tumors, hepatocellular carcinoma cells, gastric cancer cells, and colon cancer cells (121, 122). However, AE exhibits low solubility in aqueous medium (~19 μM), leading to poor oral absorption and bioavailability (123). Furthermore, long-term administration of AE may result in genotoxicity, including gene lesions and mutations, and pose potential risks such as the occurrence of acute renal failure. These factors constrain the widespread application of AE in the medical field. Consequently, research efforts aimed at enhancing the aqueous solubility of AE assume significant importance as they can substantially improve its bioavailability (124–126).

Nanomaterials are widely recognized as exceptional drug carriers due to their good biodistribution, enhanced bioavailability, and low drug toxicity. Li et al. developed AE-encapsulated nanoliposomes using reverse evaporation to improve the bioavailability of AE against human gastric cancer cells (126). Unfortunately, there have been few studies on nanomaterials for AE-mediated aPDT. AE has emerged as a promising agent for aPDT, garnering considerable attention for the treatment of surface or localized bacterial infections in recent years. Studies conducted by Li and Wang et al. provide evidence that AE-mediated aPDT is highly effective in inactivating *in vitro* isolates of MDR *Acinetobacter baumannii (A. baumannii)* and successfully treating infections caused by MDR *A. baumannii* following thermal burn injuries in mice. In summary, AE, as an exceptionally promising PS, exhibits tremendous potential in the context of managing of superficial infections caused by MDR *A. baumannii* through aPDT (65, 127). Ma et al. confirmed that AE-aPDT exhibited significant efficacy in the inactivation of *C. albicans* cells in a concentration-dependent manner by causing damage to the cell wall, cytoplasm, and nuclei (66). Additionally, the research conducted by Ma et al. demonstrated that AE is highly effective in inactivating *Trichophyton rubrum* (*T. rubrum*) microconidia in a light dose-dependent manner, exhibiting substantial inhibitory effects on the growth of *T. rubrum* (67). Cui et al. reported the *in vitro* photodynamic antimicrobial efficacy of AE on *Malassezia furfur* (*M. furfur*), a lipo-dependent yeast fungus frequently found on the skin. The findings revealed that AE-mediated aPDT demonstrated remarkable effectiveness in inactivating the fungal cells in a concentration- and light energy dose-dependent manner (68). These results suggest the potential application of AE-aPDT as a promising therapeutic option for addressing *M. furfur*-related skin conditions.

### 2.8 Celastrol

*Tripterygium wilfordii* Hook F. (*Tripterygium wilfordii*), is an ivylike vine belonging to the *Celastraceae* family, widely employed as a traditional natural medicine in Chinese traditional medicine (128). The main chemical constituents of *Tripterygium wilfordii* include diterpenoids, triterpenoids and alkaloids, with triptolide and celastrol being the most studied and clinically applied components (129). *Tripterygium wilfordii* exhibits a range of pharmacological activities, including anti-inflammatory, immunomodulatory, anticancer, and anti-rheumatic effects. As a result, it finds extensive application in the treatment of autoimmune diseases, encompassing rheumatoid arthritis and systemic lupus erythematosus (130, 131). Furthermore, *Tripterygium wilfordii* has demonstrated anticancer activity and is currently under investigation as a potential anticancer drug (128). Alam et al. conducted a study exploring the application of a natural PS derived from the medicinal plant *Tripterygium wilfordii* for aPDT. The ethanolic extract and PS-enriched fraction contained six demethylated chlorophyll derivatives as active compounds. The combined treatment of red light (660 nm) and the natural PS effectively eradicated pathogenic bacteria and fungi, particularly various skin pathogens *in vitro*. The *in vivo* efficacy and adverse reactions of aPDT were evaluated using a nematode model infected with *S. aureus* and *Streptococcus pyogenes* (69).

Celastrol is a quinone methide triterpenoid natural compound that possesses a broad range of antiviral, anti-inflammatory, and anticancer properties (132). In a previous investigation, titanium dioxide (TiO_2_) nanofibers conjugated with celastrol were employed for the treatment of HepG2 cancer cells with ultraviolet A (254 nm) (128). Caruso et al. conducted a study investigating the mechanism of action of celastrol at the active site of the main SARS-CoV-2 protease, 3CLpro, employing various techniques. Their findings suggest that celastrol could potentially serve as a PS in photodynamic therapy against SARS-CoV-2 (132, 133).

### 2.9 Riboflavin

Riboflavin, scientifically termed vitamin B2, is a water-soluble vitamin with inherent photodynamic properties. It can be found in various food sources such as dairy products (milk and cheese), meat, fish, fruits, dark green leafy vegetables, bread, grains, and grain products (134). Chemically, riboflavin comprises an isoalloxazine ring attached to a ribitol side chain and exists in two coenzyme forms: flavin mononucleotide (FMN) and flavin adenine dinucleotide (FAD). These key cofactors play a pivotal role in energy metabolism as indispensable components of oxidation-reduction enzymes, reductases, and dehydrogenases (134, 135). Riboflavin, a potent light-activated free-radical producer, exhibits absorption maxima at 270 nm, 366 nm, and 445 nm, facilitating efficient generation of ROS (135, 136).

Riboflavin plays an indispensable role in maintaining human health and has exhibited the ability to hinder the growth of a diverse spectrum of microorganisms, encompassing bacteria, viruses, fungi, and parasites, suggesting its potential as an effective antimicrobial agent (134). Its biocompatibility, nontoxic characteristics, and ROS generation capacity have attracted significant attention among researchers, particularly in the field of dentistry (135). In aPDT, riboflavin serves as both a photosensitizer and a crosslinking agent. Its multifunctional properties extend beyond reducing inflammation and eradicating microbial biofilms to preserving adhesive strength in orthodontic brackets (135, 136). Studies by Maisch et al. and Mahsa et al. have showcased the safety and effectiveness of riboflavin-based aPDT in eradicating multidrug-resistant bacteria such as *S. aureus*, *E. coli*, *P. aeruginosa*, *A. baumannii*, and *E. faecalis* biofilm. Despite the widespread use of riboflavin as a PS in aPDT, its water-soluble nature limits its incorporation rate in diverse biological tissues. Consequently, numerous studies have focused on enhancing its bioavailability by employing riboflavin derivatives or nanodelivery systems. Zhang et al. demonstrated that riboflavin formulated into a nanoemulsion exhibited potent bactericidal effects against *S. aureus* cell membranes (70, 71, 137). Additionally, Du et al. found that supramolecular materials loaded with riboflavin were capable of killing gram-positive bacteria (*e.g.*, *S. aureus*), gram-negative bacteria (*e.g.*, *E. coli*), and multidrug-resistant *S. aureus* (71). These approaches aim to overcome the challenges associated with riboflavin solubility and improve its effectiveness in aPDT.

## 3 The photochemical mechanism and targets of aPDT

aPDT relies on the generation of ROS by PSs upon exposure to specific wavelengths of light. This process involves the transfer of electrons or energy from the excited PSs to molecular oxygen (138), leading to photochemical reactions of Type I or Type II (139). In type I reactions, the excited PS transfers high-energy electrons to nearby molecules, often molecular oxygen, resulting in the production of ROS, including hydrogen peroxide (H_2_O_2_), superoxide anion (O_2_^•-^), and hydroxyl radical (·OH), among others (134, 140). Type II reactions involve the transfer of energy from the PS to oxygen, generating highly reactive singlet oxygen (^1^O_2_) (52). These two reaction types induce oxidative stress and cellular damage, ultimately leading to cell death. The equilibrium between Type I and Type II reactions can be influenced by specific substrates, PSs, and oxygen levels (141). Recently, a novel mechanism termed the “Type III photochemical pathway” has been proposed, which is an oxygen-independent mechanism for antimicrobial photoinactivation. Currently, this mechanism has been primarily observed under anaerobic/hypoxic conditions, involving PSs such as psoralens and tetracyclines, as well as the addition of organic salts such as potassium iodide and sodium azide (142, 143).

**Figure 2:**
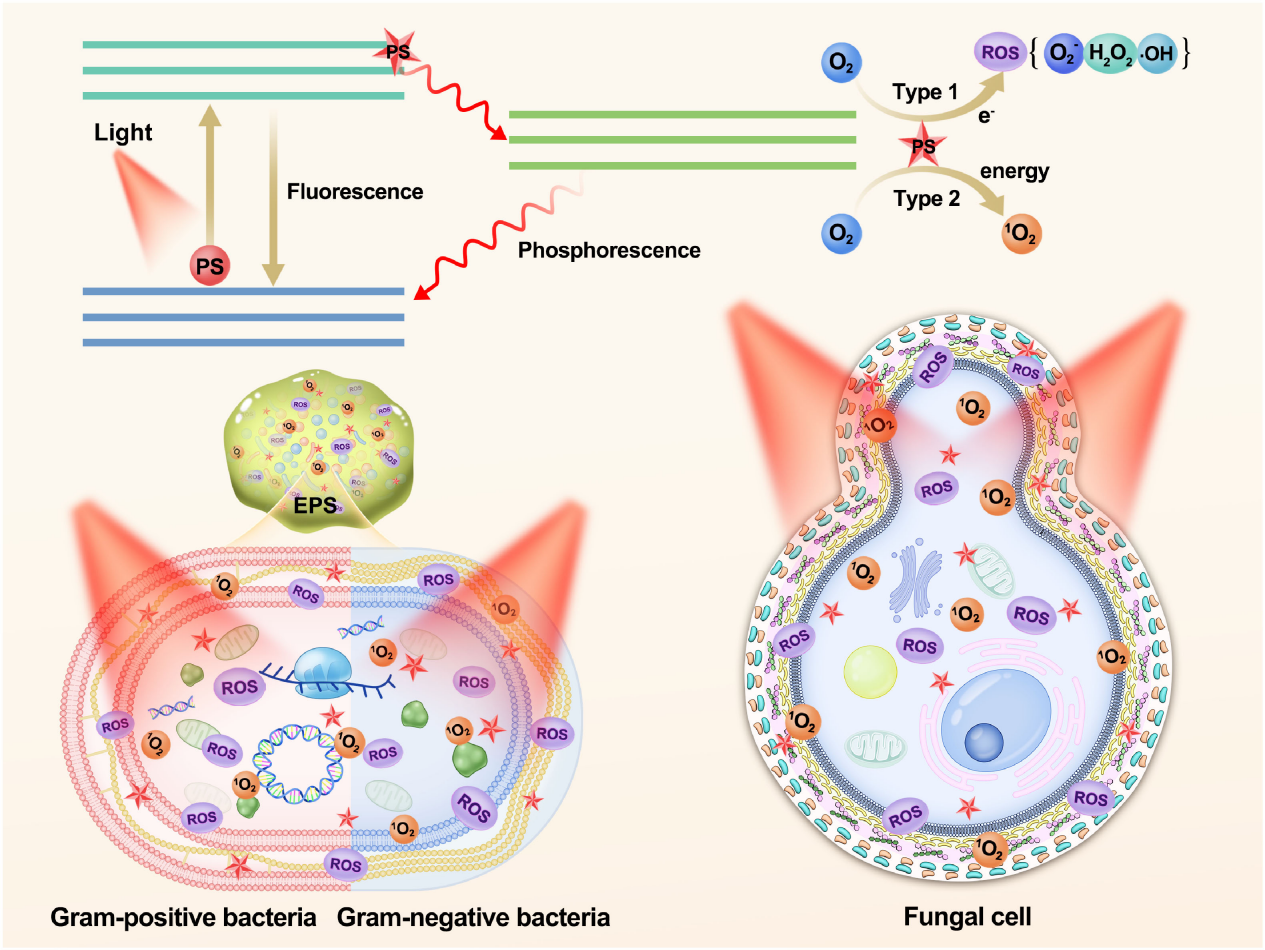
Schematic illustration of the photochemical mechanisms and the role of polyphenols as photosensitizers in aPDT targeting biofilms, Gram-positive bacteria, Gram-negative bacteria, and fungal cell. PS, photosensitizer; ROS, reactive oxygen species; EPS, extracellular polymeric substances.

aPDT is a multitarget process that inflicts damage on multiple levels. Natural product PSs can be categorized into three distinct types according to their proximity and interaction with bacterial cells: (i) PSs positioned in close proximity to the bacterial cell wall, (ii) PSs exhibiting affinity for bacterial cells, potentially causing oxidative damage to extracellular structures, and (iii) PSs capable of penetrating bacterial cells and reaching the cytoplasm, thereby exerting detrimental effects on intracellular components such as cytoplasmic proteins or DNA (144). Overall, aPDT operates through ROS generation and subsequent oxidative damage, with PSs targeting various cellular components depending on their location and interaction with bacterial cells. Understanding the mechanism and targets of aPDT is crucial for optimizing treatment strategies and developing effective antimicrobial interventions (**Figure 2**).

### 3.1 biofilm

The formation of biofilms involves the adhesion and aggregation of bacteria on living or nonliving surfaces. Biofilms exhibit a complex and organized structure, providing protection and facilitating the survival and growth of the microorganisms within the community (145, 146). They represent a distinct lifestyle from planktonic states and serve as a survival strategy for microorganisms in challenging environments (15, 147). Extracellular polymeric substances (EPS), comprising proteins, extracellular DNA (eDNA), polysaccharides, humic substances, and water-insoluble compounds, such as cellulose, amyloid proteins, nonamyloid protein fibers, and lipids, surround and immobilize biofilm cells (148). Biofilms shield microorganisms from host defense systems, increasing their tolerance to various antibiotics and disinfectants. which can result in persistent and difficult-to-treat infections (149, 150). However, polyphenolic natural product-mediated aPDT has shown significant potential in targeting biofilms and inactivating clinically relevant microorganisms. Minhaco et al. reported that curcumin-loaded PLGA nanoparticles presented effective antimicrobial activity against endodontic biofilms. Notably, encapsulated curcumin demonstrated potent antibacterial effects on both mono- and multispecies biofilms (*e.g.*, *E. faecalis, S. mutans, and Streptococcus oralis*) at a lower concentration (29). A study by Ribeiro et al. demonstrated that curcumin-mediated aPDT, when irradiated with LED light, effectively generated photoproducts, and ROS, such as singlet oxygen and free radicals, inducing phototoxicity. Thus, PDT with curcumin significantly reduced the viability of MRSA strains in biofilms (24).

Hypericin-mediated aPDT has shown effective activity against both methicillin-susceptible and methicillin-resistant *S. aureus* biofilms, as evidenced in the study conducted by García et al. (151); nevertheless, inactivation of *S. aureus* biofilms was not achieved with HYP alone, as shown in the study by Kashef et al. Interestingly, the combination of HYP with acetylcysteine exhibited remarkable efficacy in eradicating the preformed mature biofilms of *S. aureus* strains. The authors hypothesized that acetylcysteine’s ability to degrade the extracellular polysaccharide matrix of the biofilm enhances the susceptibility of biofilm-associated bacteria to the phototoxic properties of HYP (49). Xiang et al. observed that AE does not disrupt the anchoring of surface proteins to the cell wall. Instead, its inhibitory effect on biofilm development was attributed to the downregulation of specific surface protein expression or the direct obstruction of adhesion of these proteins to other matrix components (119).

### 3.2 Cell wall and cell membrane

Bacteria consist of three primary components: the cell wall, cell membrane, and cytoplasm (152). The cytoplasmic membrane shares a similar structure in both gram-negative bacteria and gram-positive bacteria, consisting of a phospholipid bilayer along with minor lipids and proteins (153). However, extensive research has demonstrated that gram-positive bacteria exhibit higher sensitivity to aPDT than gram-negative bacteria due to differences in their cell wall structures. Gram-positive cells have a single thick peptidoglycan layer surrounding their cytoplasmic membrane, resulting in higher porosity of their cell walls. Consequently, this increased porosity facilitates easier diffusion of the PSs into the intracellular space. In contrast, gram-negative bacteria possess a highly selective and complex outer membrane composed of lipopolysaccharides, lipoproteins and lipoteichoic acids, along with a thin peptidoglycan layer. These factors collectively make the penetration of PSs significantly more challenging (154, 155). In the study, Wang et al. demonstrated that quercetin had the ability to disrupt the cell wall and cell membrane structures in both gram-positive and gram-negative bacteria. This disruption increased the permeability of these structures, leading to the release of cellular cytoplasmic contents and impairment of adenosine triphosphate (ATP) activity (152). Furthermore, Lee et al. illustrated that the inactivation process of aPDT mediated by quercetin involved damage to *E. coli* O157:H7 and *L. monocytogenes* membranes through the generation of ROS. The predominant mechanism observed was type I, with O_2_^•-^ and H_2_O_2_ identified as the main ROS involved (52). The fungal cell wall consists of a cell membrane containing various membrane proteins. At the outermost layer, mannoproteins form a protective fibrous layer that conceals the underlying β-glucan layer, while chitin is situated in close proximity to the cell membrane (156, 157). In their investigation, Jan et al. discovered that HB-mediated aPDT resulted in significant impairment to the cell wall, cell membrane, cytoplasm, and nucleus of *C. albicans*, suggesting that ROS might be accountable for the damage observed in the cytoplasm and cell wall components, signifying a distinct mechanisms from that of antifungal drugs (59) (**Figure 2**).

### 3.3 Nucleic acids, proteins and lipids

To date, there have been relatively few studies investigating the direct influence of polyphenolic natural product PSs on bacterial nucleic acids, proteins, and especially lipids in aPDT. Previous research suggested that the DNA of microorganisms was primarily affected when they were either inactivated or nonviable, rendering the probability of developing resistance mechanisms against aPDT extremely low (155, 158). In a study by Lee et al., quercetin was identified as an exogenous PS located outside bacterial cells that generates ROS. This process initiated the attack on bacterial cells from the outermost structures. Subsequently, quercetin diffused into the damaged bacteria, and the ROS generated upon its entry resulted in the degradation of bacterial DNA (52). Furthermore, quercetin exhibited the ability to reduce bacterial protein synthesis, thereby affecting protein expression within the cell. Ultimately, this disruption led to cell lysis and death (152). Despite the lipid-rich composition of the bacterial cytoplasm and outer membranes, our understanding of the lipid-related mechanisms underlying natural product-mediated aPDT remains limited. The complexity associated with identifying and characterizing lipid damage has contributed to this gap (155).

## 4 *In vivo* aPDT with polyphenols

Currently, research on polyphenol-based natural product-mediated aPDT is primarily focused on oral and skin diseases in both *in vivo* (**Table 2**). In a study conducted by Dascalu Rusu LM and colleagues, utilizing curcuma extract, arnica oil, and oregano essential oil, novel natural PSs mediated aPDT effectively improved induced periodontal disease in rats and reduced inflammation (12). Paolillo FR et al. discovered that a combination of curcumin (0.06 mL of 1.5% curcumin gel) and blue light (450 nm, 80 mW/cm^2^, at the dose of 60 J/cm^2^)-mediated aPDT, with artificial skin, accelerated bacterial inactivation (*S. aureus* 4.14 log10) and enhanced wound healing in Wistar rats without inducing adverse effects on the tissue (159). Muniz IPR et al. demonstrated that *ex vivo* activation of curcumin (100 μg) by blue LED light (450 nm) at a fluence of 13.5 J/cm^2^ effectively controlled *S. aureus* cutaneous infection in type I diabetic mice (36, 160). Alam et al. achieved significant eradication of Ampicillin-Resistant *P. aeruginosa* in the *Caenorhabditis elegans* (*C. elegans*) model by using HYP in conjunction with ampicillin and subsequent orange light treatment (48). Liu et al. assessed the antibacterial capabilities of Poly (lactic acid)-Hypocrellin A (PLA-HA) nanofiber membranes through *in vivo* photodynamic therapy in rats infected with *C. albicans*. The study revealed that PLA-HA-mediated aPDT significantly promoted wound healing, reduced the infected wound area, and increased the wound healing rate by approximately 10% compared to other groups (56). Guo et al. discovered that lipase-sensitive methoxy poly (ethylene glycol)-block-poly(ϵ-caprolactone) (mPEG-PCL)/HA micelles mediated aPDT (470 nm, 90 mW/cm^2^, 60 min) effectively eradicated MRSA in the abdominal cavity of mice, increasing the survival rate to 86% at a low concentration of 10 mg/kg (HA concentration) (58). Hashimoto et al. treated burn mice infected with *P. aeruginosa* with HB: La^+3^ and aPDT (LED, 24 J/cm^2^). They found that aPDT reduced bacterial burden at the burn wound, delayed bacteremia, and lowered bacterial levels in the blood by 2-3 logarithmic units. Survival rates of mice increased 24 hours after treatment (60). Dos Santos et al. observed that blue LED light (54 J/cm^2^) enhanced the antimicrobial effect of resveratrol (2 mg/mL, 100 µL) against MRSA. In a mouse abscess model, it induced the production of TNF-α and IL-17A cytokines, reduced bacterial burden, and consequently decreased inflammation 24 hours after infection (61). Ma et al. demonstrated that AE-mediated aPDT effectively treated tinea corporis caused by *T. rubrum* in a guinea pig model and tinea unguium in an *ex vivo* model (67). *In vivo* studies reported by Wang et al. showed that AE-mediated aPDT effectively treated skin infections caused by multidrug-resistant *A. baumannii* in mice following burn injuries (127). Alam et al. evaluated the efficacy of ethanol extract of *Tripterygium wilfordii* (TWE)-mediated aPDT against various pathogens (*E. coli*, *S. aureus*, MRSA, *S. pyogenes*, and *C. albicans*) in a nematode model. Their findings indicated that it effectively controlled the pathogens without inducing strong adverse effects. TWE-mediated aPDT reversed the growth inhibition caused by pathogen infection in the nematodes, reduced the viable pathogen count associated with *C. elegans*, and improved the survival rate of the nematodes infected with *Pyogenic Streptococcus*, in conjunction with aPDT (69). Du et al. uniformly applied riboflavin G4 hydrogel (2 mL) onto sterile dressings and treated wounds infected with MRSA in rats by irradiating them with blue light at a wavelength of 460 nm and a light power density of 80 mW/cm^2^ for 10 min. Their results revealed that the hydrogel exhibited robust antimicrobial activity in the rat infection wounds after irradiation (71).

**Table 2:** *In vivo* aPDT with polyphenols.

Authors	Polyphenols	Disease Models	Effects	References
Dascalu Rusu LM et al.	CUR extract	Rats’ periodontal disease	Effectively improved periodontal disease and reduced inflammation	(12)
Paolillo FR et al.	CUR	Wistar rats wound healing	Accelerated bacterial inactivation and enhanced wound healing	(159)
Muniz IPR et al.	CUR	*S. aureus* cutaneous infection of type I diabetic mice	Effectively controlled S. aureus cutaneous infection	(36)
Galinari CB et. al.	HYP	Mouse dermatophytosis caused by *M. canis*	After three treatment, a rapid improvement in clinical symptoms at the infection site;After six treatments,a statistically significant reduction in fungal burden compared to untreated infected animals	(160)
Alam et al.	HYP	*C. elegans* of Ampicillin-Resistant *P. aeruginosa* infection	Achieved significant eradication of Ampicillin-Resistant P. aeruginosa	(48)
Liu et al.	HA	Rats infected with *C. albicans*	Significantly promoted wound healing, reduced the infected wound area	(56)
Guo et al.	HA	Mouse abdominal MRSA infection model	Effectively eradicated MRSA in the abdominal cavity of mice	(58)
Hashimoto et al.	HB	Burn mice infected with *P. aeruginosa*	Reduced bacterial burden at the burn wound, delayed bacteremia, and lowered bacterial levels	(60)
Dos Santos et al.	Resveratrol	A mouse abscess model of MRSA infection	induced the production of TNF-α and IL-17A, reduced bacterial burden, and decreased inflammation	(61)
Ma et al.	AE	Tinea corporis caused by *T. rubrum* in a guinea pig model	Effectively treated tinea corporis	(67)
Wang et al.	AE	A mouse skin infection model caused by *A. baumannii* multidrug after burn	Effectively treated skin infections	(127)
Alam et al.	*Tripterygium wilfordii*	Pathogen-infected nematode model	Effectively controlled the pathogens and improved the survival rate of the nematodes infected with *Pyogenic Streptococcus*	(69)
Du et al.	Riboflavin	A rat model of wound infection with MRSA	Exhibited robust antimicrobial activity in the rat infection wounds	(71)

CUR, curcumin; HYP, hypericin; HA, hypocrellin A; HB, hypocrellin B; AE, aloe emodin; *S. aureus*, *Staphylococcus aureus*; *M. canis*, *Microsporum canis*; *C. elegans*, *Caenorhabditis elegans*; *P. aeruginosa*, *Pseudomonas aeruginosa*; *C. albicans*, *Candida albicans*; MRSA: methicillin-resistant Staphylococcus aureus; *T. rubrum*, *Trichophyton rubrum*; *A. baumannii*, *Acinetobacter baumannii*.

## 5 Conclusions and perspectives

In recent years, aPDT has emerged as a pioneering modality specifically formulated for the inactivation of an extensive array of microorganisms, including bacteria, fungi, and viruses. Its application has grown progressively in diverse fields, notably in dermatology for conditions such as acne, in oral health for issues such as tooth decay and halitosis, and in managing fungal infections and viral diseases, notably COVID-19. Additionally, aPDT’s effectiveness in eliminating pathogens has paved its way into the food industry, bolstering food safety measures. PSs, a crucial component of aPDT, are responsible for generating ROS. Natural polyphenolic compounds derived from plants, fruits, vegetables, and other natural sources are increasingly used as PSs in aPDT due to their lower toxicity, structural diversity, and excellent biocompatibility. However, their clinical application is limited by factors such as water solubility. To overcome these limitations, innovative techniques such as nanotechnology have been employed. Nanoparticles, in particular, have proven to be efficacious drug delivery systems for hydrophobic PSs, facilitating their effective transport both *in vitro* and *in vivo*. They enable circumvention of physiological and biological barriers, thereby enhancing bacterial cell uptake. Despite these advancements, further research and technological innovation are imperative to fully exploit the potential of natural polyphenolic PSs and enhance their efficacy in treating a plethora of infectious diseases. Overcoming their limitations and achieving enhanced efficacy in the treatment of various infectious diseases will require continuous exploration and innovation.

Overall, natural polyphenolic PSs-mediated aPDT, in combination with nanoparticle-based drug delivery systems, holds great potential in combating microbial infections and advancing the field of infectious disease treatment. With concerted efforts and ongoing research, it is expected that aPDT will continue to evolve and find wider applications in the future.

## Author contributions

GH: Funding acquisition, Investigation, Writing – review & editing. XYW: Conceptualization, Visualization, Writing – original draft. RF: Writing – review & editing, Investigation. LW: Writing – original draft, Funding acquisition, Investigation. LZ: Writing – original draft, Investigation, Visualization. XJ: Funding acquisition, Writing – review & editing. XW: Conceptualization, Funding acquisition, Investigation, Writing – review & editing.
